# Dialysis Complications in AKI Patients Treated with Extended Daily Dialysis: Is the Duration of Therapy Important?

**DOI:** 10.1155/2014/153626

**Published:** 2014-08-11

**Authors:** Bianca Ballarin Albino, André Luis Balbi, Daniela Ponce

**Affiliations:** São Paulo State University (UNESP), Distrito de Rubião Junior, s/n, 18618970 Botucatu, SP, Brazil

## Abstract

This trial aimed to compare the dialysis complications occurring during different durations of extended daily dialysis (EDD) sessions in critically ill AKI patients. We included patients older than 18 years with AKI associated with sepsis admitted to the intensive care unit and using noradrenaline dose ranging from 0.3 to 0.7 *μ*g/kg/min. Patients were divided into two groups randomly: in G1, 6 h sessions were performed and, in G2, 10 h sessions were performed. Seventy-five patients were treated with 195 EDD sessions for 18 consecutive months. The prevalence of hypotension, filter clotting, hypokalaemia, and hypophosphataemia was 82.6, 25.3, 20, and 10.6%, respectively. G1 and G2 were similar in male predominance and SOFA. There was no significant difference between the two groups in hypotension, filter clotting, hypokalaemia, and hypophosphataemia. However, the group treated with sessions of 10 hours showed higher refractory to clinical measures for hypotension and dialysis sessions were interrupted more often. Metabolic control and fluid balance were similar between G1 and G2. In conclusion, intradialysis hypotension was common in AKI patients treated with EDD. There was no difference in the prevalence of dialysis complications in patients undergoing different durations of EDD.

## 1. Background

The high mortality rate among critically ill acute kidney injury (AKI) patients remains an unsolved problem in intensive care units (ICU) in spite of the considerable technological progress in renal replacement therapy (RRT) [1–3]. Dialytic management of these patients is difficult because of the associated hemodynamic instability and multiple organ dysfunction, with mortality rates reaching 50–70% [4].

There is no consensus in the literature on the best dialysis method; intermittent haemodialysis (IHD) and continuous renal replacement therapies (CRRT) have been used in AKI [5–10]. A hybrid therapy called extended daily dialysis (EDD) has emerged as an alternative to CRRT in the management of haemodynamically unstable patients with AKI [11, 12]. Its duration can range from 6 to 18 hours and it has advantages such as reduced cost, reduced need for anticoagulation, and time optimisation. The common dialysis complications in critically ill AKI patients are hypotension, coagulation system, hypokalaemia, and hypophosphataemia [13–15]. Hypotension is the most frequent complication and may occur in over 20% of AKI patients.

There are few studies in the literature on EDD in AKI patients and they involve a small number of patients [9, 13, 15–18]. They have demonstrated that EDD is well tolerated in critically ill patients, with comparable ultrafiltration (UF) and solute removal to CRRT and peritoneal dialysis [13, 16]. Regarding intradialysis hypotension, the results are controversial and different studies suggest that its prevalence ranges from 0 to 50% [9, 13–18]. This prospective clinical trial was designed to evaluate and compare the intra- and postdialysis complications in critically ill AKI patients undergoing EDD sessions lasting 6 or 10 h. We hypothesized that EDD sessions lasting 10 hours would cause less hypotension than EDD sessions lasting 6 hours.

## 2. Patients and Methods

### 2.1. Study Population

This is a prospective randomised clinical trial conducted from January 2012 to June 2013 in patients enrolled in the Brazilian University Hospital. The protocol was approved by the Institutional Ethical Committee. Written informed consent was obtained from patients or their next of kin. Patients were eligible for enrolment if they were 18 years of age or older, with AKI associated with sepsis and on a noradrenaline dose ranging from 0.3 to 0.7 *μ*g/kg/min. AKI was defined according to Acute Kidney Network criteria [19] and sepsis was defined according to Survival Sepsis 2010 [20].

Exclusion criteria were severe chronic kidney disease (basal creatinine higher than 4 mg/dL), previous chronic dialysis, kidney transplantation, and noradrenaline dose higher than 0.7 *μ*g/kg/min. These last patients were excluded because they could not tolerate actual ultrafiltration (UF) of 300–500 mL/h and, because of that, they were treated with CRRT.

### 2.2. Criteria for Initiating and Stopping EDD and Patient Randomisation

The indications for dialysis were uraemic symptoms, BUN level > 100 mg/dL (azotaemia), volume overload, electrolyte imbalance (potassium > 6 mEq/L after clinical treatment), or acid-base refractory disturbances (bicarbonate < 10 mEq/L after reposition). A patient was considered for enrolment if the judgment of the treating nephrologists was that he or she required dialysis and the mean arterial blood pressure (BP) was higher than 80 mm Hg, with a noradrenaline dose lower than 0.7 *μ*g/kg/min in the 8 hours preceding randomisation.

Patients were divided into two groups randomly, according to prescribed treatment time. Randomization was performed using sealed envelopes:

 group 1 (G1), patients undergoing EDD sessions lasting 6 hours,
 group 2 (G2), patients undergoing EDD sessions lasting 10 hours.

Dialysis was interrupted when there was partial renal function recovery (dialysis-independent) defined as restoration of urine output higher than 1000 mL/24 h associated with a progressive fall in serum values for creatinine (<4 mg/100 mL) and BUN (<50 mg/dL), a need to change dialysis method because of infectious, mechanical, or haemodynamic complications, more than 30 days of follow-up, or death.

### 2.3. Dialysis Prescription and Dialysis Complications

The EDD session lasted 6 or 10 hours according to randomisation and, for practical reasons, it was decided that EDD would be carried out 6 days a week (Monday–Saturday). Dialysis nurses and dialysis technical nursing were responsible for EDD and operated the dialysis machines throughout the treatment. A double lumen catheter for central venous access (jugular, subclavian, or femoral vein, depending on the ease of access) was inserted blindly at the bedside by nephrologists, under local anaesthesia. An HD machine with volumetric control (*Fresenius 4008F* or* Gambro K200*) and cellulose acetate dialysers (CA 150 or 170 with surface areas of 1.2 and 1.5 m^2^, resp.) were used for sessions of 6 and 10 hours, respectively. Blood flow was 200 mL/min and dialysate flow was 300 mL/min.

Anticoagulation was achieved with unfractionated heparin (usually a 1000 U bolus followed by 500 U/h) or saline flushes of 100 mL given every 30 min if anticoagulation was contraindicated. If EDD was interrupted for procedures, it was restarted later, attempting to complete 6 or 10 h of treatment. UF was prescribed during dialysis treatment as per the daily requirements. UF was performed at 300 mL/h to 500 mL/h and adjusted according to the alteration in haemodynamic parameters and fluid status of individual patients.

Bicarbonate (26 to 35 mEq/L), potassium (2 or 3 mEq/L), and sodium dialysate concentrations (142–148 mEq/L) were adjusted according to individual requirements. Dialysate temperature was low (35.5°C) to prevent hypotension.

During the procedures, BP monitoring was performed every 30 min. Hypotension was defined as a single systolic BP of less than 90 mm Hg or a mean arterial pressure (MAP) of less than 60 mm Hg. To treat a hypotension episode during EDD, protocols were applied involving the infusion of saline, discontinuation of UF, and an increased dose of vasoactive drugs, according to the clinical condition and fluid status of the patient. If, despite the measures above, haemodynamic instability persisted, posing risks to the patient, the therapy was discontinued.

Filter clotting was diagnosed as the presence of blood clots in the circuit, composed of dialyser and lines, which prevented the continuation of therapy. Hypokalaemia and hypophosphataemia were considered postdialysis complications, characterised by serum levels below 3.5 mEq/L and 3.5 mg/dL, respectively.

Treatment duration, episodes of filter clotting and replacement, vasoactive drug dose, and UF rate were recorded at the end of each session. Posttreatment BUN levels were measured by the slow flow method (with blood pump speed reduced to 50 mL/min). Blood samples were obtained from the arterial sampling port before the blood reached the dialyser. HD adequacy was determined by using urea kinetic modelling based on Kt/V [21]. The delivered dose was determined by the single-pool Kt/V value, corrected for actual UF but not for the reappearance of urea nitrogen [21]. Blood urea nitrogen, arterial blood pH, serum levels of bicarbonate, potassium, and phosphate, urine output, and fluid balance were recorded daily. Other clinical data were collected: sex, age, the presence of comorbidities (diabetes, chronic kidney disease, and hypertension), primary diagnosis, the aetiology of sepsis, prognostic score specific for AKI (ATN-ISS) [22], SOFA [23], vasoactive drug dose before and after therapy, sessions numbers, the filter used, blood and dialysate flows, and actual UF.

### 2.4. Sample Size Calculation

The sample size calculation was based on the assumption that the overall hypotension would be 50% and that a difference of 20% in hypotension between patients undergoing sessions lasting 10 and 6 hours had to be detected to be clinically relevant. With a first-order error of 5% and a power of 80% a sample size of 59 sessions was needed in each treatment group.

### 2.5. Statistical Analysis

Data analysis was performed using SAS for Windows (version 9.2: SAS Institute, Cary, NC, USA, 2012). All analyses were performed according to the intention-to-treat principle. Variables with normal distribution are described using means ± standard deviation; variables with a nonnormal distribution are described using medians and interquartile ranges. *T*-test was used to compare parametric variables between two groups. For the analysis of repeated measures, asymmetric distribution (gamma) under the GENMOD procedure was used. Multiple-comparison tests were performed by the same procedure using the DIFF option. In all statistical tests, the level of significance was 5%.

## 3. Results

**Figure 1:**
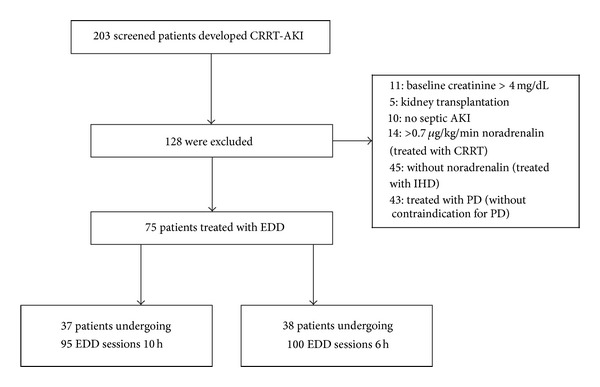
Inclusion of patients enrolled in the study.

During the study period (January 2012 to June 2013), a total of 203 patients were treated by dialysis: 101 by EDD (49.6%), 45 by conventional IHD (22%), 14 by CRRT (6.9%), and 43 by high-volume peritoneal dialysis (PD; 21.1%). The modality chosen was based on patients' haemodynamic instability. PD was indicated when there was no contraindication for its use (recent abdominal surgery, multiple abdominal surgeries, severe hyperkalaemia with electrocardiogram changes, severe respiratory failure (FiO_2_ < 70%), and severe fluid overload). Conventional IHD was indicated for haemodynamically stable patients (without vasoactive drug use). EDD was indicated when patients were using a noradrenaline dose lower than 0.7 *μ*g/kg/min and CRRT was indicated when this dose was higher than 0.7 *μ*g/kg/min. Twenty-six patients treated with EDD were withdrawn (25.7%) because of severe kidney disease (baseline creatine higher than 4 mg/dL), kidney transplantation, or AKI of other aetiologies. The remaining 75 patients were treated with 195 EDD sessions and included in the final analysis (Figure 1).

The age was 61.8 ± 15.1 years and 70.6% of patients were male. The abdomen was the main infection site (42.6%) and, among the comorbidities, hypertension was the most prevalent (49.3%). The prognostic specific to acute tubular necrosis (ATN), the ATN-ISS, was 0.69 ± 0.1, and the SOFA score was 13.6 ± 2.7. Hypotension was the main dialysis complication (82.6%), followed by filter clotting, hypokalaemia, and hypophosphataemia, which occurred in 25.3%, 20%, and 10.6% of patients, respectively.  Table 1 shows the clinical characteristics of AKI patients treated with EDD.

G1 consisted of 38 patients treated with 100 sessions, whereas G2 consisted of 37 patients treated with 95 sessions. Comparison of the clinical characteristics showed that G1 and G2 were similar in male predominance (65.7% versus 75.6%, resp., *P* = 0.34), age (63.6 ± 14.7 versus 59.9 ± 15.5 years, resp., *P* = 0.28), the presence of comorbidities such as hypertension (55.2% versus 43.2%, resp., *P* = 0.29) and diabetes (21% versus 18.9%, resp., *P* = 0.8), heart failure (52.6% versus 56.8%, *P* = 0.41), the prognosis specific to ATN (ATN-ISS) (0.68 ± 0.1 versus 0.71 ± 0.1, resp., *P* = 0.47), and SOFA score (13.1 ± 2.4 versus 14.2 ± 3.0, resp., *P* = 0.2). These results are shown in Table 2.

Table 3 shows the dialysis complications present in the two groups treated with EDD sessions of different durations. There was no significant difference between the groups in relation to intra- and postdialysis complications.

When dialysis complications were analysed by session, hypotension was present in 116 sessions (59.5%), while filter clotting was present in 29 sessions (14.9%). An increased noradrenaline dose was needed in 85.2% of the sessions and 19.1% of the sessions were interrupted. However, the group treated with sessions of 10 hours showed higher refractory to clinical measures for hypotension and dialysis sessions were interrupted more often (9.5 versus 30.1%, *P* = 0.03). The two groups were also similar regarding dialysis complications, as shown in Table 4. In G1, an increase in noradrenaline dose was needed in 53 sessions (84.1%), while it was needed in 44 sessions (83%) in G2 (*P* = 0.89). Therapy was interrupted in six sessions (9.5%) in G1 and in 16 sessions (30.1%) in G2 (*P* = 0.03).

The metabolic and fluid control of AKI patients treated with EDD lasting 6 and 10 hours are shown in Table 5. The two groups were similar in their levels of BUN, creatinine, potassium, and bicarbonate, pH, fluid balance, and actual UF. When evaluating initial and final doses of noradrenaline and BP in the first three sessions, we found that the two groups were similar and that the final dose of VAD was higher than the initial dose, which suggests that BP was maintained due to the increased dose of VAD. These data are shown in Table 6.

Table 7 shows the distribution of intradialysis complications according to duration of the sessions. The complications occurred mainly in the first session, and they were less frequent after the second or third session, which indicates improved patient tolerance to treatment.

## 4. Discussion

This clinical trial evaluated and compared intra- and postdialysis complications in critically ill AKI patients undergoing EDD sessions of different durations (6 versus 10 h). There are few studies on EDD in AKI patients and most of them included a small number of patients or are review articles, and none of them compared dialysis complications of EDD sessions of different durations [11–19].

Hypotension is the main dialysis complication in critically ill AKI patients and it may occur in over 20% of patients [14]. The decrease in BP can interrupt the dialysis treatment, leading to patients not receiving the prescribed dialysis dose, thereby affecting metabolic and fluid control. Another consequence is renal hypoperfusion, which leads to renal ischaemic injury and can delay the recovery of its function [24]. In this study, hypotension was frequent and present in 62 patients (82.6%) and 116 EDD sessions (58.9%), despite the measures to avoid hypotension, such as the low temperature of dialysate (35 to 35.5°C), high sodium (142–145 mmol/L), and actual UF rate not exceeding 500 mL/h. There was no difference between the two groups treated with EDD sessions of 6 versus 10 h in relation to hypotension episodes (63 versus 55.8%, *P* = 0.21).

To solve the problem of hypotension during EDD sessions, protocols including saline infusion, the discontinuation of UF, and an increase in noradrenaline dose were applied, according to the clinical condition of the patient and fluid status. If, despite the above measures, the instability of haemodynamics persisted, posing risks to the patient, the therapy was discontinued, which occurred in 22 sessions (19.1%).

The results of previous prospective investigations are controversial and hypotension during EDD sessions prevalence ranges from 0 to 50% [9, 13–18, 25]. Similar results were reported by Fieghen et al. [18], who evaluated haemodynamic stability in AKI septic patients treated with EDD versus CRRT. Hypotension was observed in 22 (56.4%) patients treated with EDD and in 43 (50%) patients treated with CRRT (*P* = 0.51). Ponce et al. [9] also evaluated 1367 EDD sessions of 6 or 8 h in 231 AKI patients and observed hypotension in 49.6% of the sessions. In 18.4% of them, increased inotropic support was required, and, in 19 sessions (1.4%), EDD was interrupted because of ventricular tachycardia or the increase of noradrenaline dose to higher than 1 *μ*g/kg/min.

In our study, the final noradrenaline dose was higher than the initial dose, suggesting that BP was kept stable due to the increase in noradrenaline dose. However, previous smaller prospective investigations showed that EDD was very well tolerated [11–13, 16–18, 24–26]. Berbece and Richardson [13] described 165 EDD sessions in haemodynamically unstable patients and observed hypotension in only 14% of the sessions, which was solved with the discontinuation of UF, a bolus of saline or albumin, an increase in noradrenaline dose, or interruption of the EDD session.

The two groups were similar in hypotension and increase in noradrenaline dose. We believe it happened because the group treated with sessions of 10 hours showed higher refractory to clinical measures for hypotension and dialysis sessions were interrupted more often (9.5 versus 30.1%, *P* = 0.03). The effective duration of dialysis in group 1 was 5 h 46 min and 8 h 48 min in group 2.

The results of this study indicate a higher prevalence of hypotension than that reported in the literature [9, 13–18, 25]. This can be explained by the severe haemodynamic instability and cardiovascular state of the population studied. Increasing the duration of the dialysis session to 10 hours was not an alternative to decrease number of hypotension. Other options would be to start EDD with a low UF rate and increasing it after 20–30 min to avoid the initial BP drop, considering a high initial noradrenaline dose, and treating many of these patients with CRRT.

Filter clotting is another important intradialysis complication. It involves treatment interruption and blood loss from the patient, which may contribute to the haemodynamic instability. It is characterised by staining blood extremely dark shadows or black striations in the capillary, with changes in venous and transmembrane pressures [27]. In this study, filter clotting occurred in 19 patients (25.3%) and in 29 sessions (14.9%), similar to the data reported in the literature. There was no difference in filter clotting between the groups treated with 6 versus 10 h of EDD sessions (11 versus 18.9%, *P* = 0.72).

Berbece and Richardson [13] observed filter clotting in 18% of EDD sessions conducted with heparin and 29% of heparin-free treatments. Kumar et al. [17] compared EDD sessions versus CRRT and observed that the need for anticoagulation was significantly lower in patients treated with EDD (*P* < 0.001). Kielstein et al. [11] evaluated 56 EDD sessions and observed 17 episodes of filter clotting (30%). The anticoagulation treatment in EDD sessions in our study was performed according to the comorbidities and bleeding risk of the patient, and when it was not feasible, we applied saline infusion every 30 min. In our study, 16 patients (42.1%) in G1 and 15 patients (40.5%) in G2 received anticoagulation treatment (*P* = 0.71). In terms of the sessions, 41 (41%) in G1 and 26 (27%) in G2 involved anticoagulation (*P* = 0.09).

When we evaluated intradialysis complications in the first three EDD sessions, we observed that these complications were more frequent in the first session, and, after, they became less frequent. Doshi and Murray [25] reported that intradialysis hypotension had an aetiology associated with patient characteristics, with comorbidities such as hypovolaemia, sepsis, and heart failure, and with therapies such as removal of excess fluid and reduction of serum osmolality. According to Davenport et al. [27], patients with AKI associated with sepsis had increased platelets, leukocytosis, and activation of the coagulation cascade, which could lead to filter clotting during an HD session. We believe that, after the first session, sepsis was well controlled and we could better evaluate the patient and the complications presented during therapy and thus adjust the treatment in the next sessions, such as the UF rate and heparin dose, which would explain the better patient tolerance to EDD after the first session.

Postdialysis complications were less frequent than intradialysis complications and there was no difference between the two groups regarding postdialysis complications. Hypokalaemia and hypophosphataemia occurred in 10.6 and 20% of patients, respectively. There are few studies comparing these complications after dialysis, with which to compare our results. Marshall et al. [16] analysed 145 sessions in 37 patients and showed hypokalaemia and hypophosphataemia in 7 (4.8%) and 18 (12.4%) episodes, respectively, similar to the results found in our study. Palevsky et al. [28] observed hypophosphataemia in 12.4% of patients treated with EDD in the ATN study.

Metabolic control and fluid status were similar in the groups treated with 6 h versus 10 h EDD sessions. The BUN and creatinine levels were higher in the first session in both groups and stabilised thereafter. URR was constant, around 0.6 in both groups, while delivered Kt/V was close to 1.0, actual UF remained around 2000 mL per session, without exceeding 500 mL/h and fluid balance was kept around −600 mL in both groups. Similar results have been reported in the literature. Fieghen et al. [18] evaluated 39 EDD sessions in 13 AKI patients and obtained an UF of 1915 ± 1302 mL per session. Marshall et al. [26] evaluated 56 EDD sessions lasting 8 h and observed a Kt/V of 1.43 ± 0.28. Ponce et al. [9] evaluated 1367 EDD sessions lasting from 6 to 8 hours and performed with blood and dialysate flow of 200 and 300 mL/min, respectively, and observed an UF of 2450 ± 586 mL and weekly delivered Kt/V of 5.94 ± 0.7.

Comparative study of intensive EDD (serum urea levels greater than or equal to 15 mmol/L) and standard EDD (serum urea levels between 20 and 25 mmol/L) did not show a difference in patient survival or recovery of renal function [29]. Berbece and Richardson [13] compared 11 patients treated with 209 CRRT sessions with 23 patients treated with 165 EDD sessions and reported that the weekly delivered Kt/V was higher in patients treated with EDD (CRRT: 7.1 ±2.1 versus EDD: 8.4 ± 1.8, *P* < 0.001), but there was no impact on patient survival or recovery of renal function.

Previous studies showed weekly Kt/V values for EDD of between 5.8 and 8.4 [13, 26, 29]. Dialysis dose adequacy in AKI is a subject of controversy. Several recent trials have shown that the relationship between the dose of RRT and survival is not a linear one and a weekly delivered Kt/V of 3.6 seems to be enough [28–32].

However, there is a limitation of Kt/V as a marker of efficacy for this treatment method. A study by Eloot et al. [33] showed that, despite a comparable Kt/V, the total solute removal for creatinine and urea increased with dialysis time from 4 over 6 to 8 hours, that is, better solute removal despite identical Kt/V. This was confirmed in a recent study by Schmidt et al. [34], who compared the total urea removed based on analysis of the spent collected dialysate.

Concerning patient outcome, 13.5% of patients presented renal function recovery, 8.1% of patients remained on dialysis after 30 days, and 78.4% of patients died. In this study, the mortality rate was higher than that related to previous American and European studies, which showed an in-hospital mortality rate of AKI patients treated with EDD of 50 to 62% [6, 9, 13, 15]. However, studies performed in developing countries such as Brazil and India reported a similar mortality rate [35–37]. This study included severe septic and haemodynamically unstable AKI patients presenting high ATN-ISS and SOFA (0.69 ± 0.1 and 13.6 ± 2.7, resp.), which explains the unfavourable outcome. There was no significant difference between the groups treated with EDD sessions lasting 6 versus 10 h in relation to survival or recovery of kidney function, in agreement with Palevsky et al. [28], Bellomo et al. [31], and Faulhaber-Walter et al. [30] in the trials ATN, RENAL, and HANDOUT, respectively.

Our study has several limitations. First, the small number of patients studied and the single-centre design weaken the comparison between mortality and recovery of kidney function, and the exclusion of the sickest patients (17 patients receiving a noradrenaline dose higher than 0.7 *μ*g/kg/min) may have biased the study towards a benefit for EDD. These are very important for dialysis support and the prognosis of AKI patients and further analysis will be undertaken shortly with the results of this study, such as costs, evaluation of the catabolic state of the patients, and improvement in nutritional status after each dialysis. In addition, patients should be evaluated according to different levels of prognostic score in order to define in the same range of severity; patients in both groups showed similar changes. Despite these limitations, this was the first study to evaluate dialysis complications in septic AKI patients undergoing different durations of EDD and there were enough treatment days to permit useful data for the parameters of interest to us.

Our results showed that intra- and postdialysis complications were similar between the groups treated with EDD lasting 6 versus 10 h and that the group treated with sessions of 10 hours showed higher refractory to clinical measures for hypotension and dialysis sessions were interrupted more often, with no benefit in treating AKI patients with more prolonged sessions. The findings of our study suggest that EDD lasting 6 or 10 h may provide adequate treatment for most AKI patients, achieving adequate metabolic control and net UF. However, hypotension was the most frequent complication with sessions of this dialysis method lasting 6 or 10 h and it certainly did not contribute to renal (or cardiac, brain, or gut) functional recovery. Due to the severe haemodynamic instability of the AKI patients in the present study, we questioned whether CRRT would not be the most appropriate therapy. Finally, larger studies in this area are needed to clarify the impact of EDD on patient survival and recovery of kidney function.

## Figures and Tables

**Table 1:** Clinical characteristics of AKI patients treated with EDD.

Parameters	*n* = 75
Age (years)	61.8 ± 15.1
Male gender *n* (%)	53 (70.6)
Weight (kg)	72.8 ± 20
Focus of infection *n* (%)	
Abdominal	32 (42.6)
Pulmonary	25 (33.3)
Others	18 (24)
Comorbidities *n* (%)	
Hypertension	37 (49.3)
DM	15 (20)
CKD	9 (12)
Heart failure	41 (54.6%)
ATN-ISS	0.69 ± 0.1
SOFA	13.6 ± 2.7
Dialysis complications *n* (%)	
Hypotension	62 (82.6)
Filter clotting	19 (25.3)
Hypokalemia	8 (10.6)
Hypophosphatemia	15 (20)
Patient outcome *n* (%)	
Recovery of renal function	10 (13.5)
Chronic dialysis	6 (8.1)
Death	58 (78.3)

Amounts shown in frequency, mean, standard deviation, and proportion.

AKI: acute kidney injury, EDD: extended daily dialysis, SAH: systemic arterial hypertension, DM: diabetes mellitus, CKD: chronic kidney diseases, ATN-ISS: prognostic specific to the NTA, and SOFA: sequential organ failure assessment score.

**Table 2:** Clinical characteristics of AKI patients treated with different durations of EDD.

Parameters	G1 = 6 h (*n* = 38)	G2 = 10 h (*n* = 37)	*P* value
Age (years)	63.6 ± 14.7	59.9 ± 15.5	0.28
Male gender *n* (%)	25 (65.7)	28 (75.6)	0.34
Weight (kg)	71.5 ± 20.8	74.0 ± 19.3	0.59
Infection site *n* (%)			
Abdominal	13 (34.2)	12 (32.4)	0.88
Pulmonary	12 (31.5)	20 (54.0)	0.002
Comorbidities *n* (%)			
Hypertension	21 (55.2)	16 (43.2)	0.29
Heart failure	20 (52.6)	21 (56.8)	0.41
DM	8 (21)	7 (18.9)	0.81
CKD	6 (15.7)	3 (8.1)	0.47
ATN-ISS	0.68 ± 0.1	0.71 ± 0.1	0.47
SOFA	13.1 ± 2.4	14.2 ± 3.0	0.20
Patient outcome *n* (%)			
Recovery of renal function	4 (10.5)	6 (16.6)	0.21
Chronic dialysis	4 (10.5)	2 (5.5)	0.28
Death	30 (78.9)	28 (77.7)	0.86

Amounts shown in frequency, mean, standard deviation, and proportion.

AKI: acute kidney injury, EDD: extended daily dialysis, SAH: systemic arterial hypertension, DM: diabetes mellitus, CKD: chronic kidney diseases, ATN-ISS: prognostic specific to the NTA, and SOFA: sequential organ failure assessment score.

**Table 3:** Dialysis complications of AKI patients treated with different durations of EDD.

Complications *n* (%)	G1 = 6 h (*n* = 38)	G2 = 10 h (*n* = 37)	*P* value
Hypotension	31 (81.5)	31 (83.7)	0.80
Filter clotting	9 (23.6)	10 (27)	0.73
Hypokalemia	5 (13.1)	3 (8.1)	0.71
Hypophosphatemia	7 (18.4)	8 (21.6)	0.72

Amounts shown in proportion.

AKI: acute kidney injury, EDD: extended daily dialysis.

**Table 4:** Distribution of intradialytic complications by sessions of EDD according to different duration of sessions.

Complications *n* (%)	G1 = 6 h (*n* = 100)	G2 = 10 h (*n* = 95)	*P* value
Hypotension	63 (63%)	53 (55.8%)	0.21
Filter clotting	11 (11%)	18 (18.9%)	0.72
Effective duration of dialysis	5 h 46 min	8 h 48 min	0.021

Amounts shown in proportion.

EDD: extended daily dialysis.

**Table 5:** Metabolic and fluid control of the groups in the first three sessions of EDD.

	G1 = 6 h (*n* = 100 sessions)			G2 = 10 h (*n* = 95 sessions)			*P* value
	S1 (*n* = 38)	S2 (*n* = 28)	S3 (*n* = 15)	S1 (*n* = 37)	S2 (*n* = 24)	S3 (*n* = 17)	
BUN (mg/dL)	159 ± 60	120 ± 50	105 ± 38	152 ± 69^a^	94 ± 38^b^	96 ± 37^c^	NS
BUN post (mg/dL)	64 ± 32	47 ± 17	44 ± 20	48 ± 25^a^	43 ± 20^b^	41 ± 22^c^	NS
URR	0.61 ± 0.1	0.59 ± 0.1	0.62 ± 0.1	0.68 ± 0.1^a^	0.64 ± 0.1^b^	0.69 ± 0.1^c^	NS
Kt/V	1.09 ± 0.24	1.07 ± 0.25	1.09 ± 0.25	1.26 ± 0.26^a^	1.21 ± 0.24^b^	1.28 ± 0.27^c^	NS
Cr (mg/dL)	3.8 ± 1.4	3.2 ± 1.3	2.8 ± 1.2	3.7 ± 1.3^a^	2.7 ± 0.8^b^	2.5 ± 0.6^c^	NS
K (mEq/L)	4.4 ± 0.8	4.6 ± 1	4.4 ± 0.9	4.7 ± 1^a^	4.2 ± 0.6^b^	4 ± 0.5^c^	NS
Bic (mEq/L)	17 ± 3	18.7 ± 3	19.9 ± 3.9	18.6 ± 4.2^a^	19.7 ± 7.3^b^	21 ± 2.5^c^	NS
pH	7.2 ± 0.09	7.2 ± 0.1	7.2 ± 0.09	7.2 ± 0.1^a^	7.3 ± 0.1^b^	7.3 ± 0.09^c^	NS
Presc UF (mL)	1957 ± 933	2182 ± 857	2260 ± 812	2524 ± 916^a^	2766 ± 992^b^	2611 ± 977^c^	NS
Actual UF (mL)	1731 ± 818	1967 ± 980	2146 ± 820	2332 ± 947^a^	2214 ± 1440^b^	2376 ± 1243^c^	NS
UF rate (mL/h)	288.5 ± 136	327 ± 163	357 ± 136	233 ± 94^d^	221 ± 144^e^	237 ± 124^f^	<0.05
Fluid balance (mL)	−401 ± 181	−690 ± 40	−731 ± 125	−396 ± 47^a^	−614 ± 140^b^	−652 ± 141^c^	NS

Amounts shown in mean and standard deviation.

EDD: extended daily dialysis.

BUN: blood urea nitrogen, Cr: creatinine, URR: rate reduction of urea, K: potassium, Bic: bicarbonate, and presc UF: prescribed ultrafiltration.

^a^Similar to S1 of G1.

^b^Similar to S2 of G1.

^c^Similar to S3 of G1.

^d^Different from S1 of G1.

^e^Different from S2 of G1.

^f^Different from S3 of G1.

NS: not significant (*P* > 0.05).

**Table 6:** Initial and final dose of vasoactive drugs and blood pressure according to the different duration of sessions.

	G1 = 6 h (*n* = 100 sessions)			G2 = 10 h (*n* = 95 sessions)			*P* value
	S1 (*n* = 38)	S2 (*n* = 28)	S3 (*n* = 15)	S1 (*n* = 37)	S2 (*n* = 24)	S3 (*n* = 17)	
Initial VAD	0.55 ± 0.5^d^	0.68 ± 0.6^d^	0.52 ± 0.3^d^	0.53 ± 0.2^a,d^	0.59 ± 0.4^b,d^	0.61 ± 0.4^c,d^	NS
Final VAD	0.70 ± 0.5	0.78 ± 0.7	0.64 ± 0.5	0.74 ± 0.4^a^	0.75 ± 0.6^b^	0.70 ± 0.5^c^	NS
SBP start	118 ± 23^e^	128 ± 28^e^	113 ± 27^e^	128 ± 23^a,e^	114 ± 29^b,e^	128 ± 26^c,e^	NS
DBP start	67 ± 16^f^	64 ± 16^f^	56 ± 14^f^	68 ± 15^a,f^	64 ± 18^b,f^	68 ± 13^c,f^	NS
SBP end	114 ± 26	121 ± 27	136 ± 13	124 ± 22^a^	133 ± 23^b^	123 ± 17^c^	NS
DBP end	63 ± 16	65 ± 15	69 ± 17	68 ± 15^a^	73 ± 15^b^	67 ± 9^c^	NS

Amounts shown in mean and standard deviation.

VAD: vasoactive drug, EDD: extended daily dialysis, SBP: systolic blood pressure, and DBP: diastolic blood pressure.

^a^Similar to S1 of G1.

^b^Similar to S2 of G1.

^c^Similar to S3 of G1.

^d^Different from VAD end, *P* < 0.05.

^e^Different from SBP end, *P* > 0.05.

^f^Different from DBP end, *P* > 0.05.

NS: not significant (*P* > 0.05).

**Table 7:** Distribution of episodes of intradialytic complications according to sessions and groups.

Session	Hypotension (%)		*P* value	Filter clotting (%)		*P* value
	G1 (*n* = 63)	G2 (*n* = 53)		G1 (*n* = 11)	G2 (*n* = 18)	
S1	36.5^a,c^	37.7^b^	1.0	45.4^b^	33.3^a,c^	0.7
S2	26.9^d^	18.8^d^	0.4	9.0^d^	22.2^d^	0.6
S3	17.4	20.7	0.8	9.0	16.6	0.9

Amounts shown in proportion.

^a^Similar to S2, *P* = 0.23.

^b^Different from S2 and S3, *P* < 0.05.

^c^Different from S3, *P* = 0.004.

^d^Similar to S3, *P* > 0.05.
